# The hypotension prediction index versus mean arterial pressure in predicting intraoperative hypotension: A clinical perspective

**DOI:** 10.1097/EJA.0000000000002150

**Published:** 2025-02-27

**Authors:** Santino R. Rellum, Sijm H. Noteboom, Björn J.P. van der Ster, Jaap Schuurmans, Eline Kho, Alexander P.J. Vlaar, Jimmy Schenk, Denise P. Veelo

**Affiliations:** From the Department of Anaesthesiology, Amsterdam UMC, University of Amsterdam, Amsterdam Cardiovascular Sciences, Amsterdam, The Netherlands (SRR, SHN, BJPvdS, JS, EK, JS, DPV), Department of Intensive Care, Amsterdam UMC, University of Amsterdam, Amsterdam Cardiovascular Sciences, Amsterdam, The Netherlands (SRR, SHN, JS, EK, APJV, JS), Department of Epidemiology and Data Science, Amsterdam UMC, University of Amsterdam, Amsterdam Public Health, Amsterdam, The Netherlands (JS)

## Abstract

**BACKGROUND:**

The hypotension prediction index (HPI) predicts hypotension, with randomised trials showing a significant reduction in hypotension-related metrics. However, the reliability of previous validation studies is debated, and it's unclear if mean arterial pressure (MAP) can be used interchangeably with HPI.

**OBJECTIVES:**

This study compared the effectiveness of HPI versus MAP thresholds in predicting intraoperative hypotension, focusing on three clinically relevant metrics: time from alert to event, positive predictive value (PPV), and proportion of missed hypotensive events.

**DESIGN:**

Prospective observational study conducted between 2018 and 2020.

**SETTING:**

Single-centre, academic hospital in the Netherlands.

**PARTICIPANTS:**

Adults scheduled for elective non-cardiac surgery lasting over two hours. Of the 105 eligible patients, 91 had sufficient data for analysis.

**MAIN OUTCOME MEASURES:**

The primary outcome was the time-to-hypotensive event intervals predicted by HPI popup alerts (≥85 for ≥40 s) and MAP-alerts (70–75 mmHg). Secondary analyses examined differences between these predictors regarding the PPV and missed event rates, as well as the difference in these metrics between instant HPI-85 alerts and the six MAP-alerts.

**RESULTS:**

The largest time-to-event difference was seen between HPI-85 popup and MAP-70 alerts, with a gain of 0.58 (95% confidence interval (CI), 0.57 to 0.58) min, favouring HPI. Higher MAP thresholds reduced this time difference, but worsened PPV values, with 20.5 (95% CI, 20.3 to 20.6)% at MAP-75 compared to 55.6 (95% CI, 55.4 to 55.8)% for HPI-85 popups. Missed event proportions were similar: between one to three percent. Instant HPI-85 and MAP-72 alerts showed comparable performance, but both had suboptimal PPV values around 30%. However, adding a 40-s time-dependence to MAP's alert definition levelled the differences across the three evaluated metrics, aligning more closely with HPI-85 popup alerts.

**CONCLUSIONS:**

Using HPI-85 popup alerts does not provide additional prediction time over MAP-alerts in the 70 to 75 mmHg range, but they may be preferred due to higher PPV values. Instant HPI-85 and MAP-alerts perform similarly, with MAP-72 being closest, though these alerts more frequently occur regardless of subsequent hypotension with the potential to introduce unnecessary treatment. Adding a 40-s time-dependence to MAP-alerts to match the HPI popup characteristic eliminates distinctions between prediction time and missed events, while maintaining the higher PPV. However, whether 40sec-MAP-alerts are clinically equivalent remains to be determined in prospective clinical trials.

**TRIAL REGISTRATION:**

Clinicaltrials.gov NCT03795831 on 10 January 2019.

**KEY POINTS**

- The hypotension prediction index (HPI) popup alerts may be more clinically relevant than currently available mean arterial pressure (MAP) alerts in the 70 to 75 mmHg range, as they trigger false treatment alerts less often.
- Harmonising alert definitions for HPI and MAP, requiring trends to persist for at least forty seconds, equalises their predictive performance.
- The clinical impact of harmonising alert definitions between HPI and MAP is yet to be determined.

## Introduction

The hypotension prediction index (HPI) is an alerting tool used to predict and prevent hypotension.^1^ While its impact on mortality and adverse events has not yet been adequately studied, its implementation in randomised trials has generally shown a significant reduction in hypotension-related metrics,^2–7^ as long as an adequate number of HPI alarms were followed by interventions.^8^ However, there has been debate regarding the reliability of validation studies and whether mean arterial pressure (MAP) could be used interchangeably with HPI.

Thus far, hypotension has not been eliminated in clinical practice, while its association with adverse effects remains strong.^9,10^ Although HPI has shown promising results in clinical trials, concerns have been raised about the potential overestimation of HPI's predictive ability,^11^ attributed to potential systematic selection bias in the development of HPI^1^ and subsequent validation studies.^12–19^

Furthermore, recent literature argues that HPI might be mirroring MAP,^20,21^ and that MAP changes can be used in hypotension predictors. LepMAP_0_ is one such predictor,^22^ which is a linear extrapolation of MAP resembling the deduction of an emerging trend, essentially continuing an observed change in MAP.

Given the raised concerns with HPI validation studies, we aimed to perform novel analyses that incorporated intuitive metrics relevant and directly translatable to clinical practice: time from alert to event, proportion of erroneous predictions and proportion missed events. In these analyses we make two important distinctions: in our primary and secondary outcomes we evaluated HPI and MAP parameters strictly according to their clinical appearance. We hypothesised that if the concerns were valid, the metrics achieved by HPI would not differ from those based on MAP-alerts. In exploratory analyses, we explored the addition of theoretical adjustments to MAP-alert definitions to elucidate hidden potential.

## Methods

### Ethics

This was a retrospective exploratory re-analysis of a previous investigator-initiated, prospective feasibility study conducted at the Amsterdam UMC, the Netherlands.^23^ The medical research ethics committee METC Amsterdam UMC, Amsterdam, the Netherlands (Chairperson Dr. C.L. van der Wilt) approved the study (No. W17_362) on the 28th of September 2017, which was conducted according to the Declaration of Helsinki principles and the Good Clinical Practice guidelines. Patients gave written informed consent and were included between April 2018 and October 2020. The trial was registered at ClinicalTrials.gov (NCT03795831, principal investigator: D.P. Veelo, date of registration: January 2019). A formal study protocol was not prepared for this analysis. This paper adheres to the TRIPOD+AI reporting guidelines (Supplemental 2)^24^ and reports analysed indexes according to the STARD diagram.^25^

### Patient and public involvement

Involving patients or the public in the trial design was not considered feasible.

### Study population

Adult patients (≥18 years) scheduled for elective non-cardiac surgery, preferably with a planned duration of at least two hours, requiring invasive blood pressure monitoring, were eligible. This population provides a broad blood pressure range, representative of a cohort relevant for hypotension prediction.

### Data collection

Data was collected using an EV1000 Clinical Platform (Edwards Lifesciences, Irvine, CA, USA). The dataset consists of data points every 20 s, in which an HPI value was generated every 20 s, and haemodynamic data was averaged per 20 s. The HPI values were obtained retrospectively and were not available to clinicians during the procedure.

### Hypotension and intervention definitions

For all analyses, hypotension was defined as MAP <65 mmHg lasting at least a minute until the blood pressure normalised (≥65 mmHg) for at least one minute. To ensure a fair evaluation of alerts, those superseded by the detection of blood pressure-increasing interventions were excluded. Interventions were characterised as abrupt increases in MAP values (≥5 mmHg within 20 s or ≥ 8 mmHg within 2 min) from a baseline <70 mmHg.

### Clinically available alert definitions: hypotension prediction index and mean arterial pressure

In our primary analysis, we compared HPI and MAP in predicting hypotension using their most optimal alert appearances as currently available to clinicians.

HPI, a logistic regression-based algorithm on the HemoSphere monitor, generates a hypotension probability between 0 and 100. An audible HPI-alert is produced at ≥85 (i.e., HPI-85 alert), and when sustained for 40 s, a high alert popup screen is prompted, suggesting haemodynamic intervention (i.e., HPI-85 popup alert).^26^ These HPI-85 popup alerts were prioritised in our primary analysis, as they are the alerts most likely to be sustained, aligning with the use of HPI in recent trials.^3,27^

MAP typically triggers an audible alert when the lower limit, often MAP 65 mmHg,^28^ is reached, regardless of its duration. Increasing this lower limit might provide an early warning for a decline towards hypotension. Therefore, we have evaluated every MAP threshold in the 70 to 75 mmHg range, referred to as MAP-alerts. Given the sample rate of 0.05 Hz in our dataset, a MAP decline could be evaluated every 20 s. This means that, although in line with their clinical appearances, a dissimilarity regarding time-dependence between HPI and MAP is present in the primary analysis (i.e., 40 s for HPI, 20 s for MAP).

### Theoretical alert definitions: mean arterial pressure and LepMAP_0_

Similar to the 40-s time-dependence available for HPI-alerts, such a time-dependence might be preferable and theoretically possible for MAP declines, which were evaluated in exploratory analyses.

Alternatively, the MAP change over a specific time interval could be extended into the future to extrapolate MAP the same interval forward in time. This method was introduced as LepMAP_0_.^22^ It uses two MAP values before the MAP value it attempts to predict. The first value (MAP_-n_) is selected at a specified time interval (n) before the forthcoming MAP_0_ (see Supplemental Figure 1). Then, the second value (MAP_−2n_) is selected back in time twice that distance to obtain the slope for the LepMAP_0_ prediction:


$$LepMAP_{0}=\left(2\times MAP_{-n}\right)-MAP_{-2n}$$

Since this predictor is also not clinically available, the performance was evaluated in exploratory analyses. Finally, an additional time-dependence can also be applied to LepMAP_0_ by considering an alert only if the LepMAP_0_-hypotension prediction (i.e., <65 mmHg) persists for 40 s. This allows for harmonising the alert definitions between all predictors in exploratory analyses.

### Hypotension evaluation: assignment of the classes

We distinguished between two methods for evaluating the correct detection of hypotension and non-hypotension by the predictors above: adjacency-oriented analyses and timeframe-oriented analyses. The first method, adjacency-oriented analyses, was used in our primary and secondary analyses and required an alert to activate and remain active until the onset of hypotension to be classified true positive (TP). This approach emphasises adjacency, reflecting the predictors’ design to signal emerging hypotension trend, aligning closely with clinical relevance (see Supplemental Figure 3a). Alerts that are not followed by hypotension are classified false positives (FP). Similarly, adjacency is also applied to the evaluation of non-alerts: no hypotension during a non-alert is classified as a true-negative (TN), while hypotension within this period is marked as a false-negative (FN).

In contrast, timeframe-oriented analyses deviate from the adjacency principle. They evaluate a predefined timeframe (e.g., 20 min) following an alert. If hypotension occurs within that timeframe, it is classified as a TP. The alert can fade away within this period, as it does not need to be adjacent to the hypotensive event (see Supplemental Figure 3b). If no hypotension occurs within that timeframe, it is classified FP. This method is consistent with previous validation studies that employed a forward selection analysis and was used in some of our exploratory analyses.^16,18^ Non-alerts without subsequent hypotension were labelled TN, and non-alerts followed by hypotension were classified as FN, but only if no alert started before hypotension onset.

### Performance metrics

In previous validation studies,^1,12,13,16,18^ the primary method for assessing the predictive ability of HPI has been the area under the receiver operating characteristics curve (AUC). Statistically, this is an interesting measure that aggregates the performance of a predictor across all possible thresholds. However, clinically, comparing individual thresholds between different predictors is more insightful than comparing their AUCs. Additionally, creating an AUC for MAP-alerts across a wide range would not be meaningful since values below 65 mmHg are redundant when predicting hypotension, and higher values will not be pursued given their potential harm.

To provide clinically meaningful comparisons, we prioritised three key metrics: (1) time-to-event, representing the time available to intervene based on a prediction; (2) positive predictive value, the proportion of alerts correctly indicating hypotension; (3) missed event rate, the proportion of hypotensive events missed by the predictor.

For LepMAP_0_, the time-to-event would originally be fixed to the set interval. If 1 min-LepMAP_0_ correctly indicates hypotension, the time-to-event would be one minute. Therefore, to obtain a more meaningful time-to-event, we applied a time-to-event extension as follows: in case of a correct hypotension prediction, we shifted the MAP_-n_ and MAP_-2n_ back with intervals of *x* = 20 s. These new MAP samples were used to extrapolate LepMAP_0_ up to the onset of hypotension (*n* + *x*). This process was repeated up to 20 min before hypotension or until the extended LepMAP_0_ failed to predict hypotension; this ensured that this time-to-event was always adjacent to hypotension (see Supplemental Figure 2, time-to-event of 80 s). Additionally, the time-to-event for LepMAP_0_ was also determined from each LepMAP_0_-value below 65 mmHg to the onset of hypotension in exploratory analyses.

### Sample size calculation

The sample size was based on the parent feasibility study, tripled from the required 30 patients in accordance with ISO 81060-2 standards for evaluating non-invasive sphygmomanometers.

### Study outcomes

The primary outcome was the time-to-event difference between HPI-85 popup alerts and the six MAP-alerts (i.e., decline below 70–75 mmHg thresholds) from alert to adjacent hypotensive event (i.e., adjacency-oriented analyses). Secondary outcomes included the differences in positive predictive value (PPV) and percentage of missed events between HPI-85 popup alerts and the six MAP-alerts considering adjacency. Additionally, the difference in time-to-event, PPV, and percentage of missed events were estimated between HPI-85 alerts and the six MAP-alerts using adjacency-oriented analysis.

Exploratory outcomes included the evaluation of the same three metrics (i.e., time-to-event, PPV, and missed event rate) between HPI-85 popup alerts and 1min-LepMAP_0_, 2min-LepMAP_0_, and 5min-LepMAP_0_ using the time-to-event extension. Finally, three purely theoretical comparisons explored the performances when the alert definitions between the predictors were unified. The first analysis (a) was based on a 40-s time-dependence for their alert definition in the adjacency-oriented method; the second analysis (b) also used the 40-s time-dependence for the alert definition but compared these in a 20-min timeframe-oriented method; the third analysis (c) compared every new onset of a predictor's alert using the 20-min timeframe-oriented method (see Supplemental 1 for detailed information).

In light of the possible mirror effect, MAP values from all patients were plotted for each HPI value to visualise the MAP distribution, excluding hypotension segments and MAP outliers due to applied interventions.

### Statistical analyses

Continuous data are presented as medians with interquartile [Q1 to Q3], means ± SD, or 95% confidence intervals (CI) depending on the distribution. Normality of distribution was visually assessed based on histograms and *Q−Q* plots. Null hypothesis significance testing was avoided due to potential multiple testing bias, emphasising confidence intervals for interpreting differences between paired continuous data. Categorical data are displayed as frequencies with percentages. The number of classifications per patient for each dataset was bootstrapped with 2000 iterations to retrieve the distribution. Analyses were performed using MATLAB (Version 2022b, The Mathworks Inc., Natick, MA, USA). Only patients with available waveform data were analysed; no haemodynamic data or baseline characteristics were imputed.

## Results

**Fig. 1:**
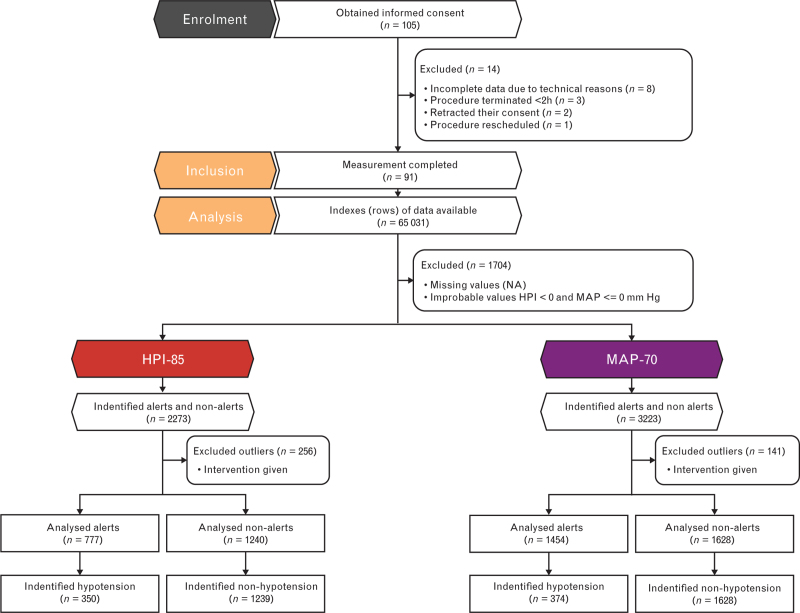
Flowchart number of participants and indexes analysed per predictor in the primary analysis.

HPI, hypotension prediction index; MAP, mean arterial pressure. These alerts refer to their clinically available forms as analysed in the primary analysis: for HPI, this means popup alerts that lasted at least 40 s, while for MAP, it refers to each new alert triggered, with values updated every 20 s.

After obtaining informed consent, 105 patients were recruited. The data from 14 patients were discarded (Fig. 1). All remaining 91 patients, without missing data were used for the analyses in this study (see Table 1 for baseline characteristics), of which 80 patients (87.9%) experienced at least one hypotensive event.

**Table 1:** Population characteristics

Category	Overall N = 91
Age (years)	63.6 ± 11.5
Sex (female)	36 (39.6)
Weight (kg)	77.3 ± 14.2
Height (cm)	174.6 ± 10.3
BMI (kg m^−2^)	25.2 ± 3.3
Atrial fibrillation	12 (13.2)
Hypertension	31 (34.1)
Diabetes Mellitus type 2	10 (11.0)
Dyslipidemia	5 (5.5)
Coronary artery disease	4 (4.4)
TIA/CVA	4 (4.4)
COPD	2 (2.2)
Smoking	
Currently	13 (14.3)
Previously	41 (45.1)
ASA score	
I	7 (7.7)
II	59 (64.8)
III	25 (27.5)
Surgical discipline	
General^a^	75 (82.4)
Neurological	8 (8.8)
Gynaecological oncology	4 (4.4)
Vascular	2 (2.2)
Colon and rectal	1 (1.1)
Oral and maxillofacial	1 (1.1)
Surgical technique	
Open	49 (53.8)
Laparoscopic	42 (46.2)
Arterial catheter (left-sided)^b^	73 (80.2)
Procedure duration (hours)	3.6 [2.7 to 4.6]

Data are presented as mean ± SD, number (%), and median [IQR]. ASA, American Society of Anesthesiologists; BMI, body mass index; COPD, chronic obstructive pulmonary disease; CVA, cerebrovascular accident; arterial catheter: invasive (radial) arterial blood pressure catheter; TIA, transient ischemic attack.

a involving mainly upper gastrointestinal and hepatopancreatic surgery

b This is the number of left-sided arterial lines in patients. The rest had right-sided arterial lines.

A total of 21 109 min of blood pressure data were analysed, containing 448 hypotensive events, amounting to 2546 minutes (12.1% of the total measuring time). A median [IQR] of 3 [2 to 8] three hypotensive events per patient, lasting a cumulative time of 14.7 [4.4 to 38.9] min, with a median area under the threshold of 49.7 [12.9 to 148.6] mmHg min^−1^ and a time-weighted-average of 0.23 [0.07 to 0.68] mmHg.

### Time-to-event differences

HPI-85 popup alerts provided a mean 0.58 min (0.57–0.58) time gain over MAP-70 alerts. While increasing the MAP threshold shifted the mean time difference to 0.34 (0.34 to 0.34) min in favour of MAP-75 (see Table 2).

**Table 2:** Primary and secondary outcomes on the three metrics between the two predictors

Prediction method		Time-to-event (min) ^a^(correctly predicted events)	Time gain for HPI-85 popup alerts (min)^a^	Time gain for HPI-85 alerts (min)^a^	% of hypotensive events missed	PPV %	NPV %
HPI-85 popup alert		2.59 (2.58 to 2.60)	−	−	1.11 (0.99 to 1.22)	55.61 (55.38 to 55.83)	100 (100 to 100)
HPI-85 alert		2.39 (2.38 to 2.40)	−	−	1.06 (0.94 to 1.17)	30.59 (30.39 to 30.78)	100 (100 to 100)
MAP alert threshold							
	70 mmHg	1.75 (1.74 to 1.76)	0.58 (0.57 to 0.58)	0.40 (0.40 to 0.41)	1.42 (1.28 to 1.57)	32.05 (31.90 to 32.19)	100 (100 to 100)
	71 mmHg	2.11 (2.10 to 2.11)	0.28 (0.28 to 0.29)	0.18 (0.17 to 0.18)	0.41 (0.33 to 0.49)	30.55 (30.42 to 30.68)	100 (100 to 100)
	72 mmHg	2.23 (2.22 to 2.24)	0.00 (0.00 to 0.00)	0.00 (0.00 to 0.00)	0.81 (0.70 to 0.93)	27.26 (27.05 to 27.47)	100 (100 to 100)
	73 mmHg	2.83 (2.82 to 2.84)	-0.00 (-0.00 to -0.00)	-0.01 (-0.01 to -0.01)	2.95 (2.75 to 3.14)	27.62 (27.42 to 27.82)	100 (100 to 100)
	74 mmHg	3.21 (3.20 to 3.22)	-0.27 (-0.28 to -0.27)	-0.32 (-0.32 to -0.32)	2.06 (1.89 to 2.23)	24.61 (24.47 to 24.75)	100 (100 to 100)
	75 mmHg	3.52 (3.51 to 3.53)	-0.34 (-0.34 to -0.34)	-0.41 (-0.42 to -0.41)	3.02 (2.82 to 3.21)	20.48 (20.31 to 20.64)	100 (100 to 100)

Data are presented as mean (95% CI). The percentage of missed events was calculated as the median proportion of correctly predicted events per patient. HPI, hypotension prediction index; MAP, mean arterial pressure; min, minute(s); NPV, negative predictive value; PPV, positive predictive value. HPI-85 popup alert results based on 40-s alert time-dependence in adjacency-oriented analyses; HPI-85 alert results based on every new alert onset in in adjacency-oriented analyses; MAP alert results based on every new alert onset in in adjacency-oriented analyses.

a The time-to-event estimates are based on correctly predicted events (missed events are not included). In contrast, time differences consider all hypotensive events. For example, if one predictor alerts 2 min before the event and another alerts 1 min before, the difference is 1 min. However, if the second predictor misses the event, the difference would be 2 min.

### Time-to-events and missed events

For HPI-85 popup alerts, the mean time-to-event was 2.59 (2.58 to 2.60) min based on the adjacency-oriented analysis; which missed 1.1 (1.0 to 1.2)% of the events, and provided a PPV of 55.6 (55.4 to 55.8)%. These metrics for HPI-85 alerts amounted to a mean time-to-event of 2.39 (2.38 to 2.40)%, 1.1 (0.9 to 1.2)% proportion of missed events and a PPV of 30.6 (30.4 to 30.8)%. MAP-75 alerts achieved a mean time-to-event of 3.52 (3.51 to 3.53) min, missing 3.0 (2.8 to 3.2)% of the events, amounting to a PPV of 20.5 (20.3 to 20.6)% (see Table 2).

**Fig. 2:**
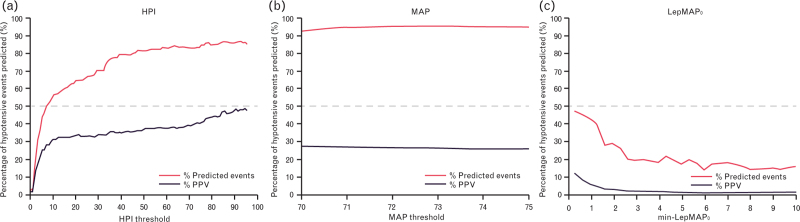
Percentage of hypotensive events preceded by an alert in the 20 min leading up to the event and the positive predictive value (PPV).

Fig. 2a to c provides a visualisation of the PPV and proportion of correctly predicted events for each of the mentioned prediction methods.

This percentage illustrates the proportion of correct predictions when assessing all events combined from all patients. For (a), the HPI values >96 are censored as these occurred primarily during hypotension.

### Exploratory outcomes

Evaluating LepMAP_0_ according to its intended use demonstrated that the 5-min time interval provided the longest time-to-event among the three intervals, achieving a mean of 5.01 (95% CI, 5.01 to 5.02) min. However, its mean proportion of missed events was 95.9 (95.7 to 96.1)%, with a mean PPV of 0.31 (0.29 to 0.32)%. Comparing 1min-LepMAP_0_ according to its intended use against HPI-85 popup alerts showed the largest difference in time-to-event, amounting to 1.99 (1.97 to 2.01) min in favour of HPI. The corresponding time-to-event estimates for the complete range of HPI and LepMAP_0_ predictions are summarised in Supplemental Tables 1 and 2.

The results of the analyses that harmonised alert definitions between predictors can be found in Supplemental Tables 3a to Supplemental Tables 3c. In the adjacency-oriented analysis, the time-to-event estimates for all predictors increased slightly when a 40-s time dependence was included. However, this increase was not observed in the timeframe-oriented analyses using the same 40-s alert definitions. Overall, applying the 40-s time dependence led to improved PPV estimates across all investigated predictors.

The comparison between the predictors after harmonising their alert definitions revealed that the differences in time-to-event, missed events, and PPV remained largely unchanged between HPI and LepMAP_0_. However, when comparing HPI popup alerts with MAP-alerts using the 40-s time-dependence, we found 40sec-MAP-72 was the most similar. This comparison showed a time-to-event difference of 0.00 (95% CI, 0.00 to 0.00) min, a mean missed event rate of 0.5 (0.4 to 0.6)%, and a PPV of 50.2 (50.0 to 50.4)%, as detailed in Supplemental Table 3a.

Supplemental Figure 5 illustrates the spread of MAP values at every HPI threshold, with the corresponding values summarised in Supplemental Table 4.

## Discussion

HPI-85 popup alerts achieved similar time-to-event provisions to MAP-alerts in the 70 to 75 mmHg range. Although the MAP-alerts occur before nearly every event, the MAP-alerts also occur without subsequent hypotension, leading to many false predictions in contrast to HPI-85 popup. Utilising 1min-LepMAP_0_ worsened the time-to-event estimations and increased the missed event rate to over 50%. Noteworthy is the inclusion of a forty-second time-dependence for MAP-alerts, which, while not (yet) clinically available, reduces false predictions and statistically levels all distinctions between HPI-85 popup alerts and 40sec-MAP-alerts.

The large proportion of missed events by LepMAP_0_ is understandable due to the often fluctuating trend of MAP. Small increases just before the onset of hypotension can translate to linear extrapolation ≥65 mmHg, highlighting the limitation of LepMAP_0_. Still, there is potential in utilising the direction of MAP changes. Consistent with the findings by Mulder *et al.*,^20^ the time-to-event estimations of MAP declines are almost identical to those of HPI-85. Further evaluation of each MAP decline revealed that most occur outside the 20 min before the hypotension, making singular declines less reliable indicators of impending events. In comparison, approximately 45% of HPI-85 popup alerts and 70% of all HPI-alerts may solicit unnecessary interventions, while intervening based on every MAP decline below 75 mmHg would result in almost 80% overtreatment. This means the HPI-85 popup alert effectively filters out false alerts, likely enhancing the effectiveness of diagnostic protocols in recent trials.^3,27^ Adding a similar time-dependence to MAP declines, in which it has to last for at least 40 s, demonstrated that the proportion of false positives can be reduced substantially as short fluctuations in MAP are filtered out. Importantly, this theoretical approach requires reconfiguring the current MAP-alert settings in available monitoring modalities. The HPI-affiliated platform presents its hypotension predictions as likelihoods, clearly conveying the urgency of an alert. In contrast, a 40 sec-MAP-alert – despite being reconfigured for improved PPV – lacks this urgency scale. A reconfigured MAP-alert should undergo the same rigorous testing as HPI, as statistical performance does not automatically ensure clinical efficacy.^29^ Furthermore, HPI evaluation could also be expanded by exploring additional alert durations to identify the optimal duration that maximises PPV without compromising event detection or intervention time. Additionally, this may incentivise manufacturers to not portray instant alerts that could contribute to alarm fatigue.

Contrary to the possible predictive similarity between HPI and MAP, Supplemental Figure 5b illustrates a range of MAP values occurring during the same HPI value. This could indicate that, despite different MAP, the algorithm recognises changes in the waveform characteristics that allow it to produce similar HPI predictions.^30^ Nevertheless, this seems in stark contrast with the strong negative association found in correlation analysis.^20^ Even adjusting for administered haemodynamic interventions and thereby removing outliers that potentially introduce higher correlation due to sudden increases in MAP and simultaneous decreases in HPI,^31^ does not substantially affect the correlation in our Spearman's rank correlation analysis. However, correlation analysis alone does not account for the temporal relation. For example, an HPI increase followed by a MAP decrease a minute later may be statistically highly correlated, but the clinically relevant temporal relation demonstrated by the delay in onset is lost. Therefore, relying solely on correlation to infer variable interchangeability is inappropriate. Nevertheless, the high correlation aligns with our exploratory analysis, where we equalised the alert definition between MAP and HPI to a 40-s threshold exceedance, showing similar time-to-event estimates.

The median time-to-event for HPI alerts, examining all thresholds, ranges from two to eight minutes, consistent with existing literature.^12,16,18^ Once more, it is important to note that HPI does not necessarily predict hypotension 5, 10, 15, and 20 min before an event. HPI was trained on blood pressure waveforms selected at those times before hypotension to recognise these events. However, these characteristics will occur gradually in anticipation of hypotension. Thus, validating HPI through a backward selection analysis, by examining a fixed time point before the onset of hypotension and non-hypotension to check if the HPI value indicated hypotension, as suggested to correct for the possible grey zone^11^ was deemed unreasonable and not performed.

Regarding the timeframe-oriented analyses, it is essential to note that in many cases, a hypotensive event occurred within 20 min after a non-alert. However, an alert often appeared before the event, still providing a timely prediction. These initial non-alerts were not considered false negatives but were discarded, in line with previous validation studies,^16,18^ increasing the sensitivity.

Our analyses are not without limitations. Without annotated data on given treatments, we filtered rapid MAP changes to correct haemodynamic interventions. However, missed interventions by this method may have overestimated the false positive proportion, and incorrectly assigned interventions may have decreased the true negative proportion. Secondly, we aimed to remove any dissimilarities between alert definitions in our exploratory analyses, but for some predictors, this meant deviating from their intended use and extending analyses to purely theoretical scenarios. Lastly, a prospective study design where each evaluated predictor is used to initiate treatment would provide a more conclusive evaluation of the actual reduction of hypotension. Ideally, once time-dependence is configurable.

In conclusion, HPI-85 popup alerts do not provide substantial time gain over MAP-alerts in the 70 to 75 mmHg range when confining to their current clinical availability. However, using these MAP-alerts as treatment triggers will introduce two times more false positives as they also occur briefly regardless of subsequent hypotension. Despite MAP's declining trend before every hypotensive event, it scarcely produces valid LepMAP_0_ predictions. While a 40-s time-dependence for MAP-alerts theoretically yields similar clinical performance, HPI is currently the only clinically available and well researched tool for predicting hypotension.

## Supplementary Material

Supplemental Digital Content

## Supplementary Material

Supplemental Digital Content
