# Supplementary material for: The hypotension prediction index versus mean arterial pressure in predicting intraoperative hypotension: A clinical perspective
Source: Eur J Anaesthesiol. 2025 Feb 27;42(6):527–35. doi: 10.1097/EJA.0000000000002150 (PMC12052080; doi:10.1097/EJA.0000000000002150)
Supplement: Supplemental Digital Content [file ejanet-42-527-s001.docx]

# Appendix 2. Supplemental materials

The Hypotension Prediction Index versus Mean Arterial Pressure in Predicting Intraoperative Hypotension: A Clinical Perspective

The authors have provided this supplemental appendix to give readers additional information about their work.

## Legend

Supplemental Figure 1. page 3

Supplemental Figure 2. page 4

Supplemental Figure 3a and 3b. page 5

Supplemental Figure 4. page 6

Supplemental Figure 5. page 7

Supplemental Table 1. page 9

Supplemental Table 2. page 10 and 11

Explanation modified alert definitions page 12

Supplemental Table 3a to 3c. page 13 to 15

Supplemental Table 4 page 16 to 18

Supplemental Table 5 page 19

# Supplemental Figures


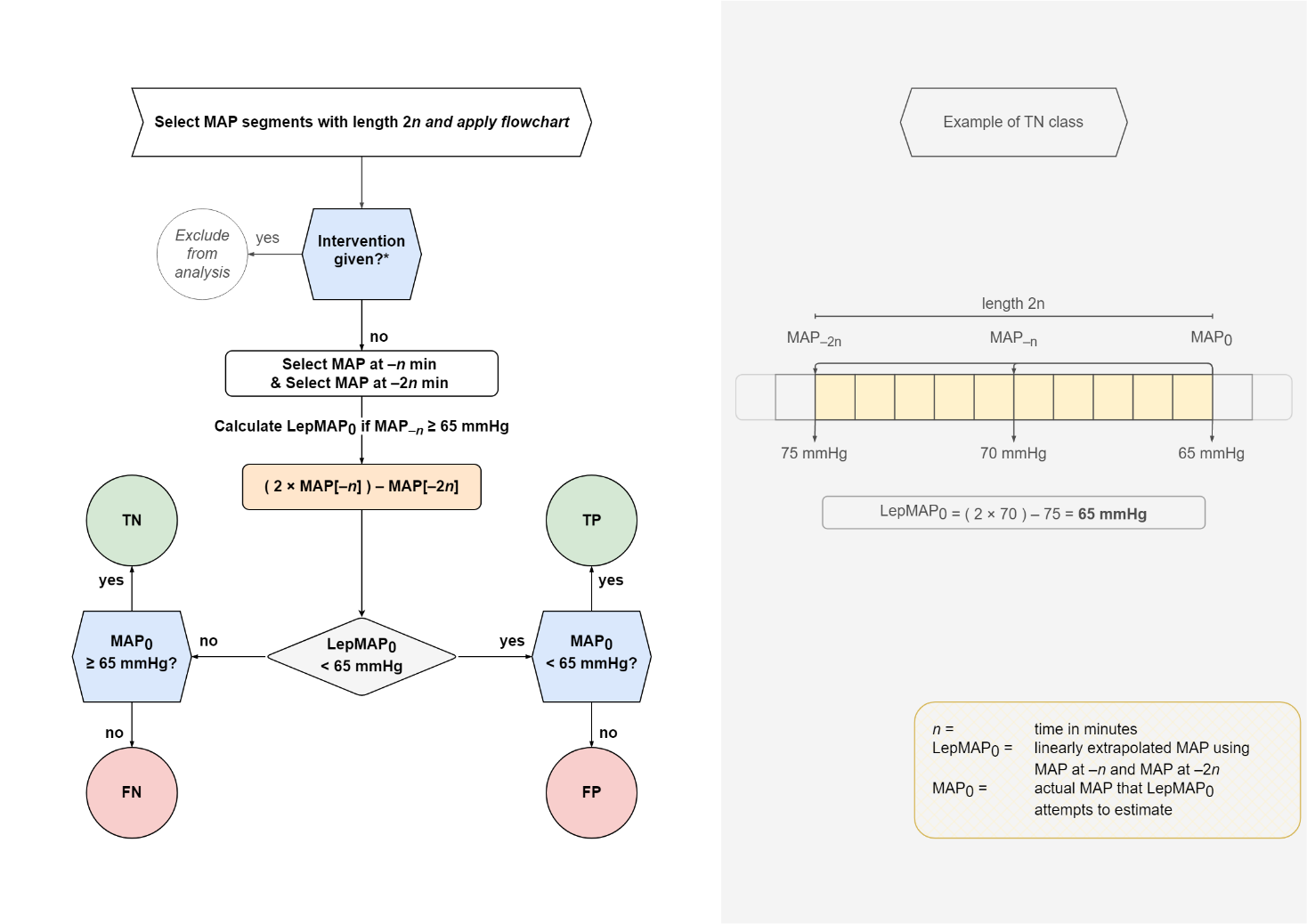


**Supplemental Figure 1. LepMAP_0_ classification flowchart**

FN: false-negative; FP: false-positive; HPI: Hypotension Prediction Index; MAP: mean arterial pressure; min: minute(s); n: time in minutes; TN: true-negative; TP: true-positive;

LepMAP_0_: linearly extrapolated MAP using MAP at –*n* and MAP at –2*n*

MAP_0_: actual MAP that LepMAP_0_ attempts to estimate

*hemodynamic intervention is assumed in case of a MAP change ≥ 5 mm Hg within 20s or ≥ 8 mm Hg within 2 min from a baseline MAP < 70 mmHg.


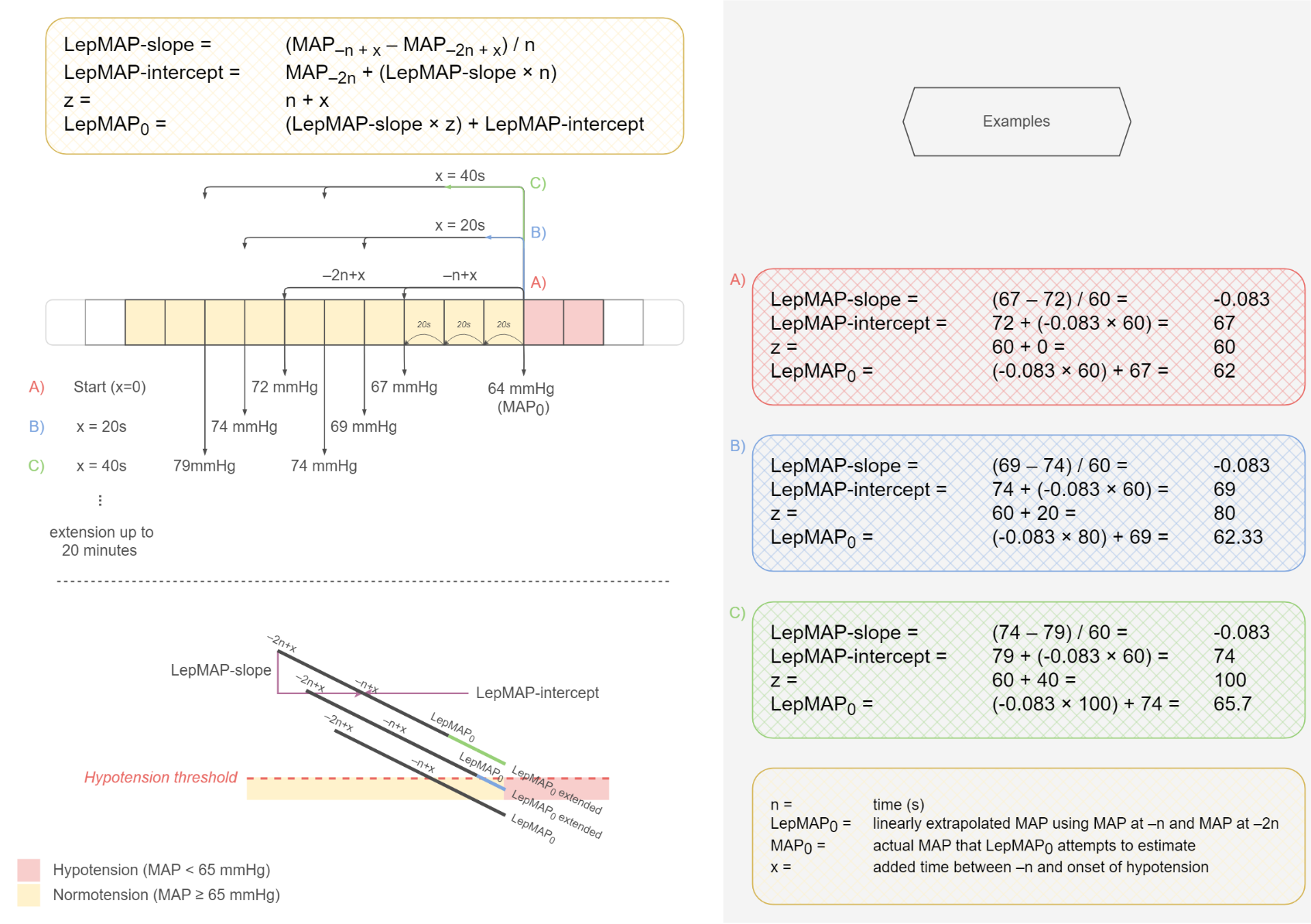


**Supplemental Figure 2. LepMAP_0_ time-to-event calculation**

MAP: mean arterial pressure; n: time in s; LepMAP0: linearly extrapolated MAP using MAP at –n and MAP at –2n

MAP0: actual MAP that LepMAP0 attempts to estimate.


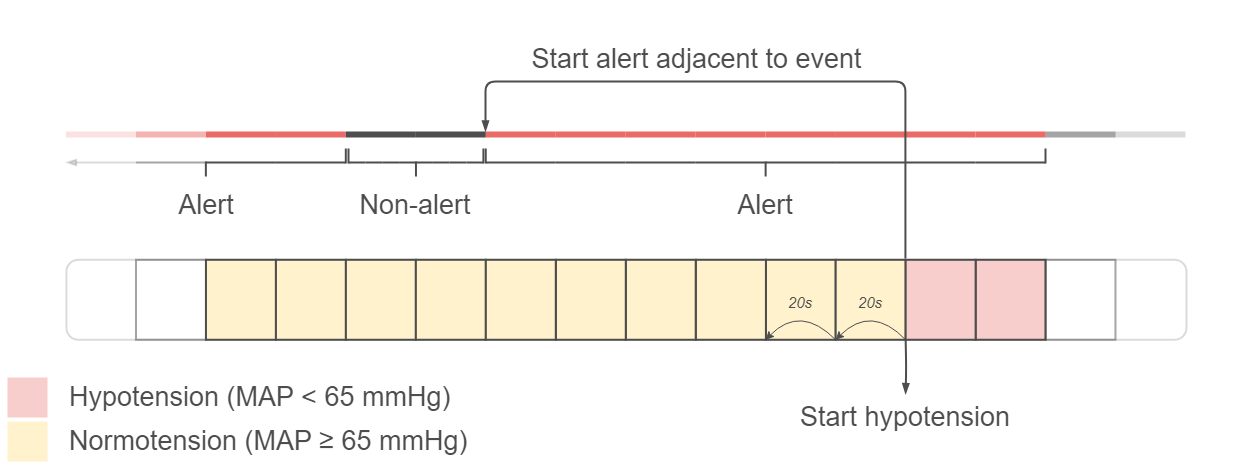


**Supplemental Figure 3a. Time-to-event evaluation in adjacency-oriented analysis**

MAP: mean arterial pressure;

**
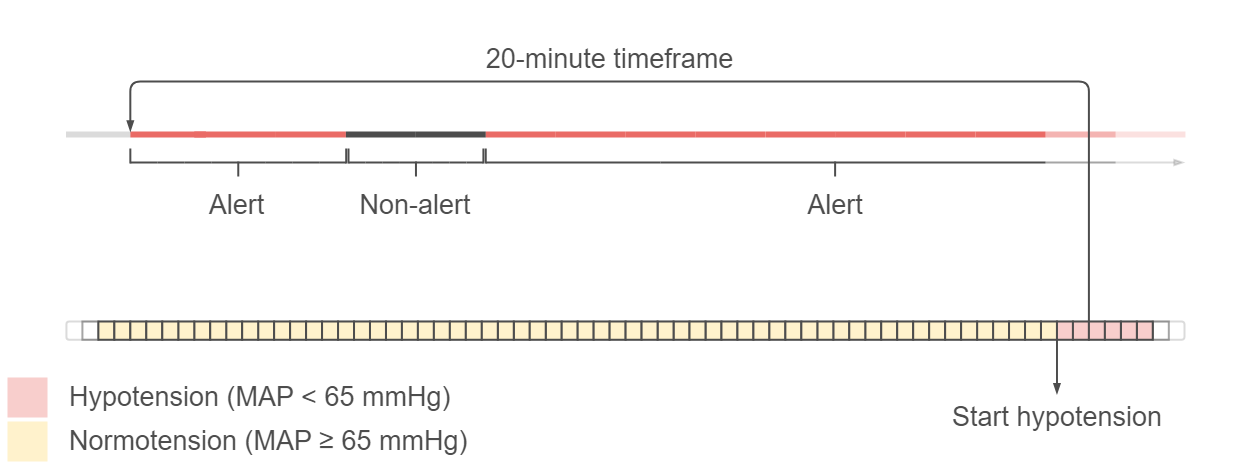
**

**Supplemental Figure 3b. Time-to-event evaluation in timeframe-oriented analysis**

MAP: mean arterial pressure;


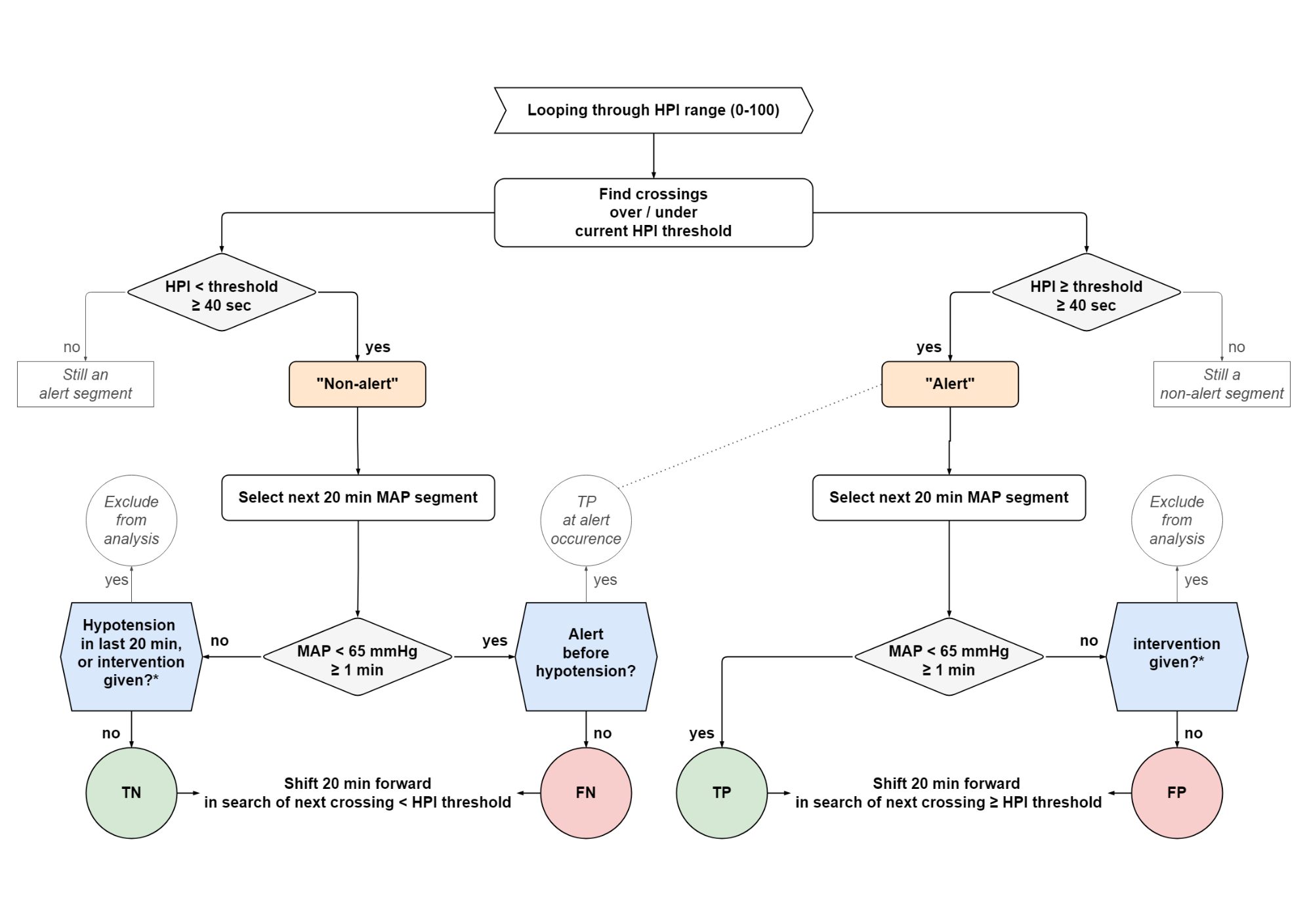


**Supplemental Figure 4. HPI classification flowchart evaluating the following 20 minutes, timeframe-oriented analysis**

FN: false-negative; FP: false-positive; HPI: Hypotension Prediction Index; MAP: mean arterial pressure; min: minute(s); TN: true-negative; TP: true-positive;

*hemodynamic intervention is assumed in case of a MAP change ≥ 5 mm Hg within 20s or ≥ 8 mm Hg within 2 min from a baseline MAP < 70 mmHg.


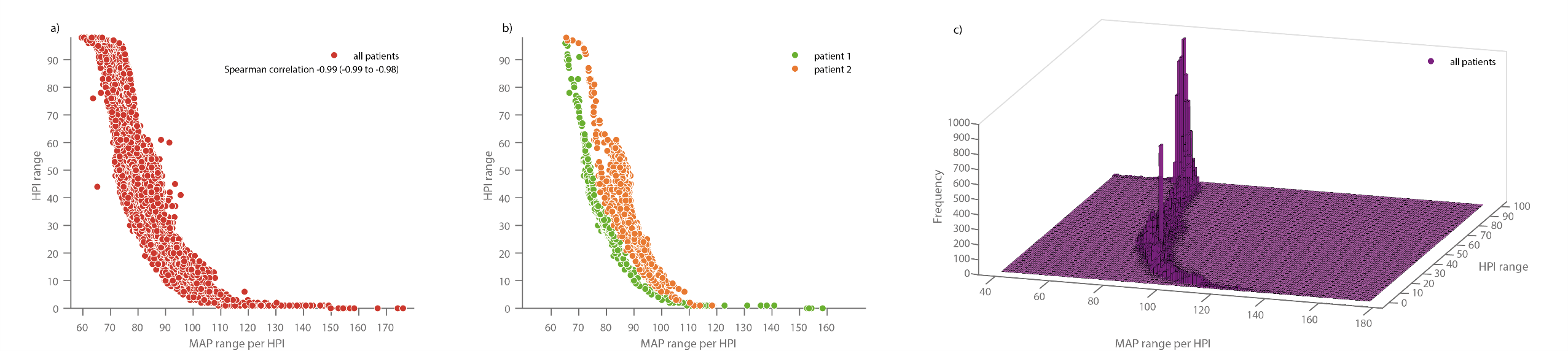

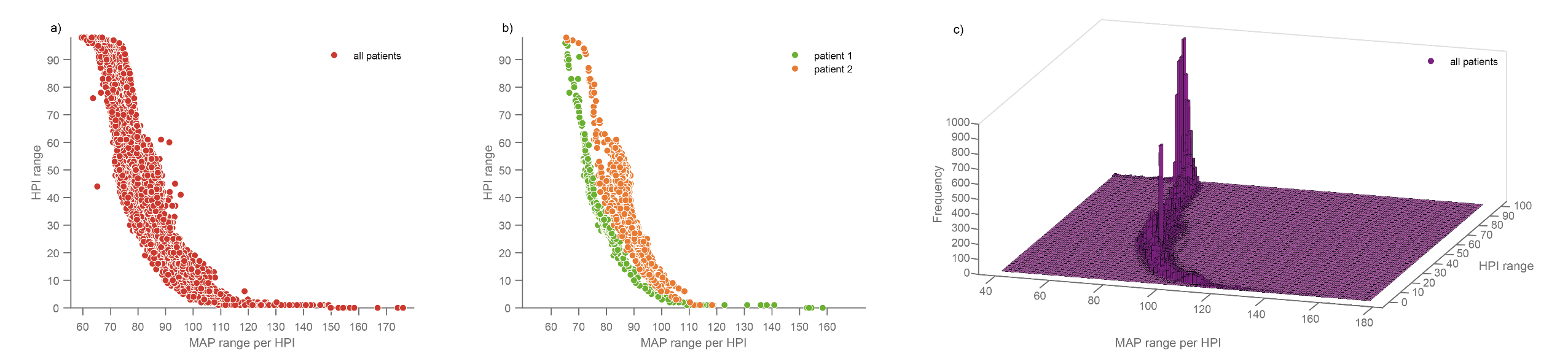


**Supplemental Figure 5. Distribution of MAP values per HPI threshold**

HPI: Hypotension Prediction Index; MAP: mean arterial pressure.

*Spearman's rank correlation between HPI and MAP, excluding hypotensive segments and given interventions, yielded a median coefficient of -0.99 (-0.99 to -0.98) based on all patients.*

# Supplemental Tables

| HPI threshold | Time-to-event*  (*correctly predicted events*) | % missed hypotensive events | PPV % | NPV % | MAP value at the onset of HPI alert  (correctly *predicted events*) |
| --- | --- | --- | --- | --- | --- |
| 5 | 3.79 (3.69 to 3.89) | 75.20 (74.58 to 75.83) | 13.03 (12.72 to 13.35) | 69.75 (67.74 to 71.76) | 77.65 (77.58 to 77.72) |
| 10 | 8.12 (8.09 to 8.14) | 45.88 (45.56 to 46.20) | 28.76 (28.59 to 28.92) | 100 (100 to 100) | 79.01 (78.94 to 79.09) |
| 15 | 8.30 (8.28 to 8.32) | 41.00 (40.74 to 41.26) | 32.35 (32.16 to 32.54) | 100 (100 to 100) | 78.54 (78.47 to 78.61) |
| 20 | 7.84 (7.83 to 7.86) | 32.88 (32.67 to 33.09) | 34.90 (34.72 to 35.08) | 100 (100 to 100) | 79.28 (79.17 to 79.39) |
| 25 | 7.19 (7.18 to 7.20) | 30.45 (30.26 to 30.63) | 34.05 (33.88 to 34.21) | 100 (100 to 100) | 79.17 (79.12 to 79.23) |
| 30 | 7.22 (7.19 to 7.24) | 27.95 (27.73 to 28.16) | 33.47 (33.31 to 33.63) | 100 (100 to 100) | 79.25 (79.20 to 79.30) |
| 35 | 6.86 (6.84 to 6.88) | 15.38 (15.10 to 15.67) | 37.10 (36.95 to 37.25) | 100 (100 to 100) | 78.97 (78.94 to 79.00) |
| 40 | 5.69 (5.66 to 5.72) | 16.95 (16.68 to 17.22) | 36.17 (36.02 to 36.31) | 100 (100 to 100) | 76.81 (76.79 to 76.83) |
| 45 | 5.54 (5.51 to 5.56) | 14.33 (14.06 to 14.60) | 38.33 (38.16 to 38.50) | 100 (100 to 100) | 75.73 (75.71 to 75.74) |
| 50 | 4.55 (4.52 to 4.57) | 12.16 (11.82 to 12.51) | 40.82 (40.60 to 41.04) | 100 (100 to 100) | 74.95 (74.93 to 74.96) |
| 55 | 4.46 (4.42 to 4.49) | 9.81 (9.45 to 10.16) | 44.71 (44.46 to 44.97) | 100 (100 to 100) | 74.29 (74.27 to 74.30) |
| 60 | 4.10 (4.08 to 4.13) | 8.43 (8.08 to 8.78) | 44.95 (44.66 to 45.25) | 100 (100 to 100) | 74.15 (74.13 to 74.16) |
| 65 | 3.59 (3.57 to 3.61) | 6.72 (6.42 to 7.01) | 42.81 (42.57 to 43.05) | 100 (100 to 100) | 73.33 (73.32 to 73.34) |
| 70 | 3.37 (3.35 to 3.38) | 6.72 (6.43 to 7.01) | 42.89 (42.62 to 43.16) | 100 (100 to 100) | 72.59 (72.58 to 72.61) |
| 75 | 3.00 (2.99 to 3.02) | 3.59 (3.34 to 3.83) | 46.79 (46.59 to 46.98) | 100 (100 to 100) | 72.02 (72.02 to 72.03) |
| 80 | 2.74 (2.72 to 2.75) | 4.82 (4.58 to 5.05) | 54.02 (53.73 to 54.31) | 100 (100 to 100) | 71.36 (71.35 to 71.37) |
| 85 | 2.59 (2.58 to 2.60) | 1.11 (0.99 to 1.22) | 55.61 (55.38 to 55.83) | 100 (100 to 100) | 70.56 (70.55 to 70.57) |
| 90 | 2.26 (2.25 to 2.27) | 1.00 (0.90 to 1.10) | 54.95 (54.74 to 55.16) | 100 (100 to 100) | 69.73 (69.72 to 69.74) |
| 95 | 2.00 (1.99 to 2.01) | 3.89 (3.67 to 4.12) | 52.58 (52.38 to 52.79) | 100 (100 to 100) | 68.31 (68.30 to 68.32) |

**Supplemental Table 1. Performance metrics for entire HPI threshold range**

Data are presented as mean (95% CI).

Time-to-event as calculated from the last onset of a HPI-85 popup alert (40-second) prior to the onset of hypotension (adjacency-oriented analysis);

HPI: Hypotension Prediction Index; min: minute(s); MAP: mean arterial pressure.

| LepMAP_0_ prediction timeframe (n) | Time gain for HPI 85 alerts (min)  (*including missed events*) | Time-to-event  (*correctly predicted events*) | % missed hypotensive events | PPV %* | NPV % |
| --- | --- | --- | --- | --- | --- |
| 0.33 min | 2.11 (2.10 to 2.12) | 0.44 (0.44 to 0.44) | 54.17 (53.99 to 54.35) | 11.40 (11.35 to 11.45) | 100 (100 to 100) |
| 0.67 min | 2.04 (2.03 to 2.06) | 0.86 (0.86 to 0.86) | 55.22 (55.02 to 55.42) | 7.76 (7.71 to 7.81) | 100 (100 to 100) |
| 1.00 min | 1.99 (1.97 to 2.01) | 1.20 (1.19 to 1.20) | 59.90 (59.74 to 60.06) | 5.81 (5.78 to 5.84) | 100 (100 to 100) |
| 1.33 min | 1.64 (1.63 to 1.66) | 1.38 (1.37 to 1.38) | 63.95 (63.88 to 64.03) | 4.59 (4.57 to 4.62) | 100 (100 to 100) |
| 1.67 min | 1.75 (1.73 to 1.76) | 1.71 (1.70 to 1.71) | 72.31 (72.19 to 72.42) | 2.96 (2.94 to 2.98) | 100 (100 to 100) |
| 2.00 min | 1.64 (1.64 to 1.65) | 2.12 (2.11 to 2.12) | 69.29 (69.13 to 69.45) | 2.71 (2.69 to 2.73) | 100 (100 to 100) |
| 2.33 min | 1.64 (1.63 to 1.64) | 2.53 (2.52 to 2.54) | 76.14 (75.98 to 76.30) | 1.96 (1.96 to 1.97) | 100 (100 to 100) |
| 2.67 min | 1.89 (1.88 to 1.90) | 2.86 (2.85 to 2.86) | 82.62 (82.45 to 82.78) | 1.47 (1.46 to 1.49) | 100 (100 to 100) |
| 3.00 min | 1.59 (1.58 to 1.59) | 3.16 (3.15 to 3.17) | 80.17 (80.04 to 80.31) | 1.55 (1.54 to 1.56) | 100 (100 to 100) |
| 3.33 min | 1.68 (1.67 to 1.68) | 3.49 (3.49 to 3.50) | 82.85 (82.72 to 82.97) | 1.40 (1.39 to 1.41) | 100 (100 to 100) |
| 3.67 min | 1.61 (1.61 to 1.62) | 3.75 (3.75 to 3.76) | 83.45 (83.33 to 83.57) | 1.32 (1.32 to 1.33) | 100 (100 to 100) |
| 4.00 min | 1.68 (1.68 to 1.69) | 4.14 (4.14 to 4.15) | 86.26 (86.12 to 86.41) | 1.16 (1.16 to 1.17) | 100 (100 to 100) |
| 4.33 min | 1.64 (1.63 to 1.64) | 4.35 (4.35 to 4.36) | 78.90 (78.73 to 79.08) | 1.31 (1.30 to 1.32) | 100 (100 to 100) |
| 4.67 min | 1.87 (1.86 to 1.88) | 4.73 (4.72 to 4.73) | 86.78 (86.58 to 86.97) | 1.13 (1.12 to 1.14) | 100 (100 to 100) |
| 5.00 min | 1.85 (1.84 to 1.86) | 5.01 (5.01 to 5.02) | 95.89 (95.70 to 96.08) | 0.31 (0.29 to 0.32) | 100 (100 to 100) |
| 5.33 min | 1.80 (1.79 to 1.80) | 5.38 (5.37 to 5.38) | 89.37 (89.25 to 89.49) | 0.91 (0.90 to 0.92) | 100 (100 to 100) |
| 5.67 min | 1.80 (1.79 to 1.81) | 5.70 (5.70 to 5.71) | 91.19 (90.99 to 91.38) | 0.67 (0.66 to 0.69) | 100 (100 to 100) |
| 6.00 min | 1.77 (1.76 to 1.77) | 6.00 (6.00 to 6.01) | 95.40 (95.21 to 95.59) | 0.36 (0.34 to 0.37) | 100 (100 to 100) |
| 6.33 min | 1.75 (1.74 to 1.75) | 6.35 (6.35 to 6.36) | 94.00 (93.75 to 94.24) | 0.57 (0.55 to 0.59) | 100 (100 to 100) |
| 6.67 min | 1.76 (1.75 to 1.77) | 6.70 (6.70 to 6.70) | 93.61 (93.36 to 93.87) | 0.59 (0.57 to 0.61) | 100 (100 to 100) |
| 7.00 min | 1.75 (1.74 to 1.76) | 7.00 (7.00 to 7.00) | 94.36 (94.06 to 94.67) | 0.37 (0.35 to 0.38) | 100 (100 to 100) |
| 7.33 min | 1.77 (1.76 to 1.78) | 7.42 (7.42 to 7.43) | 89.17 (89.02 to 89.33) | 0.77 (0.76 to 0.78) | 100 (100 to 100) |
| 7.67 min | 1.84 (1.83 to 1.86) | 7.67 (7.67 to 7.67) | 92.36 (92.16 to 92.57) | 0.59 (0.58 to 0.61) | 100 (100 to 100) |
| 8.00 min | 1.84 (1.83 to 1.85) | 8.01 (8.00 to 8.01) | 95.31 (95.13 to 95.50) | 0.33 (0.32 to 0.35) | 100 (100 to 100) |
| 8.33 min | 1.86 (1.85 to 1.87) | 8.34 (8.33 to 8.34) | 97.31 (97.13 to 97.50) | 0.18 (0.17 to 0.20) | 100 (100 to 100) |
| 8.67 min | 1.96 (1.95 to 1.97) | 8.68 (8.68 to 8.69) | 98.34 (98.22 to 98.47) | 0.13 (0.12 to 0.14) | 100 (100 to 100) |
| 9.00 min | 2.05 (2.04 to 2.06) | 9.03 (9.02 to 9.03) | 96.85 (96.69 to 97.01) | 0.23 (0.22 to 0.24) | 100 (100 to 100) |
| 9.33 min | 2.18 (2.17 to 2.19) | 9.34 (9.34 to 9.34) | 94.92 (94.76 to 95.08) | 0.38 (0.37 to 0.39) | 100 (100 to 100) |
| 9.67 min | 2.21 (2.20 to 2.23) | 9.68 (9.67 to 9.68) | 91.57 (91.42 to 91.72) | 0.60 (0.59 to 0.61) | 100 (100 to 100) |
| 10.00 min | 2.20 (2.19 to 2.21) | 10.01 (10.01 to 10.02) | 93.73 (93.56 to 93.91) | 0.44 (0.43 to 0.45) | 100 (100 to 100) |

**Supplemental Table 2. Performance metrics for multiple LepMAP_0_ prediction time intervals**

Data are presented as mean (95% CI).

LepMAP’s time-to-event: calculated using the extension method for correct LepMAP_0_ hypotension predictions.

HPI’s time-to-event: calculated from the last onset of a HPI-85 popup alert (40-second) prior to the onset of hypotension (adjacency-oriented analysis).

*Hypotension prediction (LepMAP_0_ < 65 mmHg).

HPI: Hypotension Prediction Index; LepMAP_0_: linear extrapolation of MAP; MAP: mean arterial pressure.

## Explanation of additional modified alert definitions

The aim of these modified analyses is to establish uniformity in the definitions across all predictors, with the consequence of negating the clinical appearance, while highlighting potential predictive capabilities if alert settings were to be changed in the available monitoring modalities. The results from these modifications are summarised for the complete cohort in the Tables 3a to 3d. For clarification, only LepMAP_0_ values that were calculated based on a MAP_-n_ value above the hypotension threshold are used for assignment of true and false positive classes.

**40-second alert time-dependence using adjacency-oriented analysis (a)**

For all three predictors an alert is defined as the exceedance of the threshold for at least 40 seconds and lasts until the exceedance is absent for 40 seconds. These alerts are classified true positive if during the segment of the alert a hypotensive event is detected within 20 minutes. If the segment lasts longer than 20 minutes, the next classification of either a true or false positive, could be assigned after a 20-minute washout period starting from the last detected hypotensive event, or 20 minutes from the last false positive classification if the alert was still active by then. Alerts are only valid when detected outside of a hypotensive event and when an intervention to increase the blood pressure is absent within the evaluated timeframe.

**40-second alert time-dependence using 20-minute timeframe-oriented analysis (b)**

For all three predictors an alert is defined as the exceedance of the threshold for at least 40 seconds and lasts until the exceedance is absent for 40 seconds. These alerts are classified true positive if in the following 20 minutes a hypotensive event is detected. Alerts are only valid when detected outside of a hypotensive event and when an intervention to increase the blood pressure is absent within the evaluated timeframe (see Supplemental Figure 4). Non-alerts without subsequent hypotension are categorized as TN, while non-alerts followed by hypotension are classified as FN, but only if no alert occurred before the onset of hypotension.

**Every new alert onset using 20-minute timeframe-oriented analysis (c)**

For all three predictors an alert is defined as every new exceedance of the threshold and lasts until the last exceedance. These alerts are classified true positive if in the following 20 minutes a hypotensive event is detected. Alerts are only valid when detected outside of a hypotensive event and when an intervention to increase the blood pressure is absent within the evaluated timeframe. Non-alerts without subsequent hypotension are categorized as TN, while non-alerts followed by hypotension are classified as FN, but only if no alert occurred before the onset of hypotension.

| Prediction method | | Time-to-event (min)* *(correctly predicted events)* | Time gain for HPI 85 alerts (min)* | % of hypotensive events missed | PPV % | NPV % | Mean alert value^ⴕ^ | |
| --- | --- | --- | --- | --- | --- | --- | --- | --- |
|  |  |  |  |  |  |  | **TP** | **FP** |
| HPI-85 | | 2.59 (2.58 to 2.60) | - | 1.11 (0.99 to 1.22) | 55.61 (55.38 to 55.83) | 100 (100 to 100) | 92.5 | 88.8 |
| LepMAP_0_ | |  |  |  |  |  |  |  |
|  | 1-min^‡^ | 0.33 (0.33 to 0.33) | 2.23 (2.22 to 2.25) | 74.50 (74.35 to 74.65) | 18.45 (18.32 to 18.59) | 78.25 (78.16 to 78.34) | 62.0 | 62.4 |
|  | 2-min^‡^ | 0.48 (0.47 to 0.48) | 2.12 (2.11 to 2.13) | 84.41 (84.26 to 84.57) | 9.49 (9.38 to 9.60) | 80.42 (80.30 to 80.54) | 60.3 | 61.7 |
|  | 5-min^‡^ | 0.71 (0.69 to 0.73) | 2.38 (2.37 to 2.39) | 88.13 (87.81 to 88.45) | 6.91 (6.74 to 7.08) | 76.44 (76.33 to 76.55) | 58.4 | 60.5 |
| MAP threshold | |  |  |  |  |  |  |  |
|  | 70 mmHg | 1.95 (1.94 to 1.95) | 0.42 (0.42 to 0.43) | 0.83 (0.71 to 0.95) | 54.25 (54.04 to 54.46) | 100 (100 to 100) | 67.3 | 68.9 |
|  | 71 mmHg | 2.29 (2.28 to 2.31) | 0.14 (0.13 to 0.15) | 0.19 (0.14 to 0.25) | 52.75 (52.58 to 52.92) | 100 (100 to 100) | 68.1 | 69.8 |
|  | 72 mmHg | 2.51 (2.50 to 2.52) | 0.00 (-0.00 to 0.00) | 0.51 (0.42 to 0.61) | 50.22 (50.03 to 50.42) | 100 (100 to 100) | 68.9 | 70.6 |
|  | 73 mmHg | 2.91 (2.90 to 2.92) | -0.00 (-0.00 to -0.00) | 0.27 (0.21 to 0.34) | 49.73 (49.54 to 49.92) | 100 (100 to 100) | 69.6 | 71.7 |
|  | 74 mmHg | 3.30 (3.28 to 3.31) | -0.28 (-0.29 to -0.28) | 0.29 (0.22 to 0.36) | 45.59 (45.39 to 45.78) | 100 (100 to 100) | 70.5 | 72.5 |
|  | 75 mmHg | 3.97 (3.96 to 3.99) | -0.37 (-0.37 to -0.37) | 4.04 (3.81 to 4.28) | 40.49 (40.28 to 40.70) | 100 (100 to 100) | 71.4 | 73.3 |

**Supplemental Table 3a. Results based on 40-second alert time-dependence in adjacency-oriented analyses**

Data are presented as mean (CI 95%) or median [Q1-Q3].

The percentage of missed events was calculated as the median proportion of correctly predicted events per patient.

*The time-to-event estimates are based on correctly predicted events (missed events are not included). In contrast, time differences consider all hypotensive events. For example, if one predictor alerts 2 minutes before the event and another alerts 1 minute before, the difference is 1 minute. However, if the second predictor misses the event, the difference would be 2 minutes.

^ⴕ^This value represents the mean output of the respective predictor during the first 40 seconds of each new alert. For instance, for MAP-75 alerts, an alert is detected if the MAP declines below 75 mm Hg for at least one minute, the average value during that first minute was recorded in each patient. The mean value corresponding to true positives and false positives are separately summarised in the table.

^‡^Hypotension prediction threshold (LepMAP_0_ < 65 mmHg).

FP: false positive; HPI: Hypotension Prediction Index; MAP: mean arterial pressure; min: minute(s); NPV: negative predictive value; PPV: positive predictive value; TP: true positive.

| Prediction method | | Time-to-event (min)* *(correctly predicted events)* | Time gain for HPI 85 alerts (min)* | % of hypotensive events missed | PPV % | NPV % | Mean alert value^ⴕ^ | |
| --- | --- | --- | --- | --- | --- | --- | --- | --- |
|  |  |  |  |  |  |  | **TP** | **FP** |
| HPI-85 | | 5.28 (5.26 to 5.29) | - | 0.25 (0.20 to 0.31) | 81.11 (80.91 to 81.30) | 100 (100 to 100) | 91.6 | 89.2 |
| LepMAP_0_ | |  |  |  |  |  |  |  |
|  | 1-min^‡^ | 4.91 (4.87 to 4.95) | 1.32 (1.31 to 1.33) | 27.38 (27.15 to 27.61) | 82.05 (81.85 to 82.24) | 100 (100 to 100) | 62.2 | 62.0 |
|  | 2-min^‡^ | 6.47 (6.46 to 6.49) | 1.33 (1.32 to 1.34) | 27.46 (27.35 to 27.58) | 71.14 (70.94 to 71.35) | 100 (100 to 100) | 61.2 | 61.3 |
|  | 5-min^‡^ | 8.62 (8.60 to 8.65) | 1.40 (1.39 to 1.42) | 34.91 (34.72 to 35.10) | 56.11 (55.74 to 56.48) | 100 (99.99 to 100) | 59.8 | 60.4 |
| MAP threshold | |  |  |  |  |  |  |  |
|  | 70 mmHg | 4.49 (4.46 to 4.52) | 0.32 (0.32 to 0.33) | 0.18 (0.13 to 0.23) | 81.64 (81.42 to 81.86) | 100 (100 to 100) | 67.6 | 68.9 |
|  | 71 mmHg | 5.25 (5.23 to 5.28) | 0.03 (0.03 to 0.03) | 0.01 (-0.00 to 0.01) | 83.08 (82.93 to 83.23) | 100 (100 to 100) | 68.5 | 69.8 |
|  | 72 mmHg | 6.06 (6.03 to 6.08) | 0 | 0.12 (0.08 to 0.16) | 81.89 (81.73 to 82.05) | 100 (100 to 100) | 69.3 | 70.8 |
|  | 73 mmHg | 6.32 (6.30 to 6.35) | 0 | 0.07 (0.04 to 0.11) | 79.77 (79.59 to 79.96) | 100 (100 to 100) | 70.0 | 71.6 |
|  | 74 mmHg | 6.66 (6.63 to 6.68) | -0.35 (-0.35 to -0.34) | 0.14 (0.10 to 0.18) | 72.93 (72.71 to 73.15) | 100 (100 to 100) | 70.9 | 72.4 |
|  | 75 mmHg | 7.67 (7.64 to 7.70) | -0.71 (-0.71 to -0.70) | 3.14 (2.92 to 3.36) | 68.36 (68.04 to 68.69) | 100 (100 to 100) | 71.9 | 73.2 |

**Supplemental Table 3b. Results based on 40-second alert time-dependence in 20-minute timeframe-oriented analyses**

Data are presented as mean (CI 95%) or median [Q1-Q3].

The percentage of missed events was calculated as the median proportion of correctly predicted events per patient.

*The time-to-event estimates are based on correctly predicted events (missed events are not included). In contrast, time differences consider all hypotensive events. For example, if one predictor alerts 2 minutes before the event and another alerts 1 minute before, the difference is 1 minute. However, if the second predictor misses the event, the difference would be 2 minutes.

^ⴕ^This value represents the mean output of the respective predictor during the first 40 seconds of each new alert. For instance, for MAP-75 alerts, an alert is detected if the MAP declines below 75 mm Hg for at least one minute, the average value during that first minute was recorded in each patient. The mean value corresponding to true positives and false positives are separately summarized in the table.

^‡^Hypotension prediction threshold (LepMAP_0_ < 65 mmHg).

FP: false positive; HPI: Hypotension Prediction Index; MAP: mean arterial pressure; min: minute(s); NPV: negative predictive value; PPV: positive predictive value; TP: true positive.

| Prediction method | | Time-to-event (min) **(correctly predicted events)* | Time gain for HPI 85 alerts (min)* | % of hypotensive events missed | PPV % | NPV % | Mean alert value^ⴕ^ | |
| --- | --- | --- | --- | --- | --- | --- | --- | --- |
|  |  |  |  |  |  |  | **TP** | **FP** |
| HPI-85 | | 6.31 (6.29 to 6.33) | - | 0.10 (0.06 to 0.13) | 69.86 (69.62 to 70.11) | 100 (100 to 100) | 90.0 | 88.6 |
| LepMAP_0_ | |  |  |  |  |  |  |  |
|  | 1-min^‡^ | 6.18 (6.16 to 6.20) | 1.03 (1.02 to 1.03) | 6.33 (6.07 to 6.58) | 76.02 (75.83 to 76.22) | 100 (100 to 100) | 62.3 | 62.0 |
|  | 2-min^‡^ | 7.28 (7.26 to 7.29) | 0.89 (0.88 to 0.91) | 14.66 (14.45 to 14.87) | 66.50 (66.22 to 66.78) | 100 (100 to 100) | 61.8 | 62.1 |
|  | 5-min^‡^ | 8.40 (8.38 to 8.43) | 1.04 (1.02 to 1.05) | 20.78 (20.60 to 20.96) | 52.99 (52.68 to 53.30) | 100 (100 to 100) | 61.6 | 61.9 |
| MAP threshold | |  |  |  |  |  |  |  |
|  | 70 mmHg | 6.05 (6.03 to 6.07) | 0.34 (0.33 to 0.34) | 0 | 76.32 (76.11 to 76.53) | 100 (100 to 100) | 68.6 | 68.9 |
|  | 71 mmHg | 6.98 (6.96 to 7.00) | 0 | 0.00 (0.00 to 0.01) | 75.05 (74.82 to 75.29) | 100 (100 to 100) | 69.6 | 70.0 |
|  | 72 mmHg | 6.74 (6.72 to 6.76) | 0 | 0.01 (-0.01 to 0.02) | 71.03 (70.80 to 71.26) | 100 (100 to 100) | 70.5 | 70.9 |
|  | 73 mmHg | 6.89 (6.88 to 6.90) | -0.01 (-0.01 to -0.01) | 0.15 (0.10 to 0.20) | 66.34 (66.04 to 66.63) | 100 (100 to 100) | 71.4 | 71.9 |
|  | 74 mmHg | 6.98 (6.96 to 7.01) | -0.26 (-0.27 to -0.25) | 0.11 (0.07 to 0.15) | 57.79 (57.39 to 58.19) | 100 (100 to 100) | 72.3 | 72.8 |
|  | 75 mmHg | 7.58 (7.54 to 7.61) | -0.53 (-0.54 to -0.53) | 0.33 (0.25 to 0.41) | 59.31 (58.90 to 59.72) | 100 (100 to 100) | 73.2 | 73.7 |

**Supplemental Table 3c. Results based on every new alert onset in 20-minute timeframe-oriented analyses**

Data are presented as mean (CI 95%) or median [Q1-Q3].

The percentage of missed events was calculated as the median proportion of correctly predicted events per patient.

*The time-to-event estimates are based on correctly predicted events (missed events are not included). In contrast, time differences consider all hypotensive events. For example, if one predictor alerts 2 minutes before the event and another alerts 1 minute before, the difference is 1 minute. However, if the second predictor misses the event, the difference would be 2 minutes.

^ⴕ^This value represents the mean output of the respective predictor during the onset each new alert. For instance, for MAP-75 alerts, an alert is detected each time the MAP declines below 75 mm Hg, the first value during that decline was recorded in each patient. The mean value corresponding to true positives and false positives are separately summarized in the table.

^‡^Hypotension prediction threshold (LepMAP_0_ < 65 mmHg).

HPI: Hypotension Prediction Index; MAP: mean arterial pressure; min: minute(s); NPV: negative predictive value; PPV: positive predictive value.

| Prediction method | | TP | FP | TN | FN |
| --- | --- | --- | --- | --- | --- |
|  |  |  |  |  |  |
| HPI-85 | | 350 | 381 | 1087 | 1 |
| LepMAP_0_ | | | | | |
|  | 1-min^*^ | 108 | 443 | 892 | 245 |
|  | 2-min^*^ | 91 | 576 | 882 | 259 |
|  | 5-min^*^ | 82 | 654 | 808 | 255 |
| MAP threshold | | | | | |
|  | 70 mmHg | 367 | 372 | 1145 | 0 |
|  | 71 mmHg | 369 | 392 | 1180 | 0 |
|  | 72 mmHg | 357 | 415 | 1166 | 0 |
|  | 73 mmHg | 353 | 444 | 1164 | 0 |
|  | 74 mmHg | 345 | 457 | 1123 | 0 |
|  | 75 mmHg | 331 | 501 | 1108 | 0 |

**Supplemental Table 4a. Results based on 40-second alert time-dependence in adjacency-oriented analyses**

^*^Hypotension prediction threshold (LepMAP_0_ < 65 mmHg).

FN: false negative; FP: false positive; HPI: Hypotension Prediction Index; MAP: mean arterial pressure; min: minute(s); TN: true negative; TP: true positive.

| Prediction method | | TP | FP | TN | FN* |
| --- | --- | --- | --- | --- | --- |
|  |  |  |  |  |  |
| HPI-85 | | 556 | 224 | 552 | 0 |
| LepMAP_0_ | | | | | |
|  | 1-min^ⴕ^ | 524 | 125 | 523 | 5 |
|  | 2-min^ⴕ^ | 480 | 191 | 538 | 5 |
|  | 5-min^ⴕ^ | 433 | 280 | 535 | 18 |
| MAP threshold | | | | | |
|  | 70 mmHg | 627 | 189 | 570 | 0 |
|  | 71 mmHg | 634 | 206 | 577 | 0 |
|  | 72 mmHg | 603 | 242 | 583 | 0 |
|  | 73 mmHg | 578 | 266 | 581 | 0 |
|  | 74 mmHg | 551 | 287 | 570 | 0 |
|  | 75 mmHg | 550 | 296 | 540 | 0 |

**Supplemental Table 4b. Results based on 40-second alert time-dependence in 20-minute timeframe-oriented analyses**

*False negatives are not counted when the alert did occur before hypotension onset.

^ⴕ^Hypotension prediction threshold (LepMAP_0_ < 65 mmHg).

FN: false negative; FP: false positive; HPI: Hypotension Prediction Index; MAP: mean arterial pressure; min: minute(s); TN: true negative; TP: true positive.

| Prediction method | | TP | FP | TN | FN* |
| --- | --- | --- | --- | --- | --- |
|  |  |  |  |  |  |
| HPI-85 | | 810 | 437 | 613 | 0 |
| LepMAP_0_ | | | | | |
|  | 1-min^ⴕ^ | 1027 | 309 | 440 | 0 |
|  | 2-min^ⴕ^ | 959 | 414 | 546 | 0 |
|  | 5-min^ⴕ^ | 786 | 554 | 642 | 7 |
| MAP threshold | | | | | |
|  | 70 mmHg | 974 | 399 | 565 | 0 |
|  | 71 mmHg | 953 | 453 | 632 | 0 |
|  | 72 mmHg | 917 | 503 | 684 | 0 |
|  | 73 mmHg | 847 | 528 | 705 | 0 |
|  | 74 mmHg | 782 | 566 | 735 | 0 |
|  | 75 mmHg | 761 | 566 | 724 | 0 |

**Supplemental Table 4c. Results based on every new alert onset in 20-minute timeframe-oriented analyses**

*False negatives are not counted when the alert did occur before hypotension onset.

^ⴕ^Hypotension prediction threshold (LepMAP_0_ < 65 mmHg).

FN: false negative; FP: false positive; HPI: Hypotension Prediction Index; MAP: mean arterial pressure; min: minute(s); TN: true negative; TP: true positive.

| HPI threshold | Mean MAP | Standard deviation | Minimal MAP | Maximal MAP | Number of MAP values per threshold |
| --- | --- | --- | --- | --- | --- |
| 0-4 | 112.6 | 9.1 | 98.6 | 176.0 | 1315 |
| 5-9 | 100.8 | 3.2 | 90.7 | 118.6 | 1525 |
| 10-14 | 95.5 | 2.7 | 86.6 | 107.9 | 1791 |
| 15-19 | 91.6 | 2.2 | 82.6 | 103.7 | 2096 |
| 20-24 | 88.9 | 2.1 | 81.7 | 98.8 | 2291 |
| 25-29 | 86.6 | 2.1 | 78.1 | 95.2 | 3101 |
| 30-34 | 83.6 | 2.3 | 76.8 | 93.2 | 2858 |
| 35-39 | 81.1 | 2.4 | 74.5 | 93.5 | 2736 |
| 40-44 | 79.6 | 2.6 | 73.4 | 95.5 | 1939 |
| 45-49 | 78.9 | 2.7 | 71.8 | 93.4 | 1615 |
| 50-54 | 78.3 | 2.8 | 71.5 | 87.8 | 1435 |
| 55-59 | 77.6 | 2.5 | 72.1 | 85.8 | 1486 |
| 60-64 | 76.5 | 2.0 | 71.0 | 91.4 | 1630 |
| 65-69 | 75.3 | 1.5 | 70.3 | 80.6 | 1264 |
| 70-74 | 74.4 | 1.5 | 69.2 | 79.3 | 1256 |
| 75-79 | 73.7 | 1.4 | 63.7 | 78.6 | 1260 |
| 80-84 | 73.1 | 1.4 | 67.3 | 77.6 | 1556 |
| 85-89 | 72.3 | 1.4 | 66.0 | 77.2 | 1850 |
| 90-94 | 71.0 | 1.5 | 65.1 | 75.7 | 3191 |
| 95-99 | 68.0 | 1.9 | 59.6 | 74.2 | 6786 |

**Supplemental Table 5. Range of MAP values per HPI threshold with standard deviation**

HPI: Hypotension Prediction Index; MAP: mean arterial pressure.

Hypotension segments are not included. Hypotension was defined as MAP values below 65 mm Hg for at least one minute.
